# Re-encountering the phobic cue within days after a reconsolidation intervention is crucial to observe a lasting fear reduction in spider phobia

**DOI:** 10.1038/s41380-024-02882-1

**Published:** 2025-01-09

**Authors:** Jacqueline Peters, Anna I. Filmer, Johnny B. van Doorn, Vivian N. Metselaar, Renée M. Visser, Merel Kindt

**Affiliations:** Department of Psychology, University of Amsterdam, Amsterdam, The Netherlands

**Keywords:** Psychology, Psychiatric disorders, Neuroscience

## Abstract

Memory reconsolidation interventions offer an exciting alternative to exposure treatment because they may target fear memories directly, thereby preventing relapse. A previous reconsolidation intervention for spider fear abruptly reduced avoidance behaviour, whereas changes in self-reported fear followed later. In this pre-registered placebo-controlled study, we first aimed to conceptually replicate these effects in spider phobia. Second, we investigated whether re-encountering the phobic cue after the reconsolidation intervention is necessary for changes in self-reported fear to occur. Third, we tested whether the window to trigger such changes is time limited. Individuals with spider phobia (*N* = 69) were randomized into three groups and underwent a memory reactivation procedure with a tarantula, followed immediately by propranolol (reconsolidation intervention) or placebo. One reconsolidation intervention group and the placebo group re-encountered spiders two days after treatment in behavioural approach tasks, whereas another reconsolidation intervention group re-encountered spiders after four weeks. Changes in spider avoidance behaviour and self-reported fear were followed for one year. In the short term, the reconsolidation intervention was not more effective than placebo: both conditions benefited from the intervention. In the long term, the reconsolidation intervention was more effective than placebo, but only when the phobic stimulus was re-encountered within days after treatment. Specifically, we found less tarantula avoidance behaviour and self-reported fear over the course of one year when spiders were re-encountered two days after the reconsolidation intervention, but not when the behavioural test was conducted four weeks after the intervention. These findings challenge the idea that a reconsolidation-inspired intervention alone is sufficient to treat clinical fears: Experiencing the behavioural change during the re-encounter within days after the reconsolidation window has closed seems crucial to observe a lasting fear reduction.

## Introduction

Exposure is a core component of Cognitive Behavioural Therapy (CBT) for anxiety disorders [1, 2], which is currently considered the gold standard in therapy [3]. However, many people relapse after initially successful treatment [1, 2, 4, 5]. The dominant model of exposure treatment suggests that a novel, fear-inhibiting memory trace is formed during exposure that competes with the older, maladaptive fear memory, which can resurface over time, when highly stressed, or when encountering the feared situation outside of the treatment context [1, 5, 6]. This can potentially lead to relapse. Memory reconsolidation interventions are an exciting alternative to exposure therapy because they are assumed to directly modify the affective value of a fear memory and could thereby prevent relapse [7–10]. Yet, results regarding the translational value of reconsolidation interventions in (sub-)clinical phobias are mixed [7, 11–13]. We aimed to better understand the mechanism of change in reconsolidation interventions by shedding light on the dissociation between the different aspects of fear memory expression that was previously observed after reconsolidation interference [8, 9, 14].

The memory reconsolidation hypothesis suggests that an established memory can be reactivated under specific conditions, causing it to return to a time-limited labile state in which it requires de novo protein synthesis for restabilization [15, 16, but see 17]. While the memory is labile, it can be targeted with pharmacological agents [18] to alter reconsolidation. This process, referred to as reconsolidation interference, is thought to directly weaken the (affective value of the) fear memory [10, 19]. As a result, there is an abrupt change in the defensive response (e.g., avoidance behaviour) when confronted with the feared situation after a night of sleep [9, 10, 20]. In contrast, a gradual decline of avoidance behaviour is typically observed over the course of multiple exposure therapy sessions [1, 21], after maladaptive thoughts and threat expectations have been challenged [22, 23]. Thus, the reconsolidation hypothesis suggests that interfering with reconsolidation weakens fear memories through a fundamentally different mechanism than exposure treatment: Instead of forming a new inhibitory memory that competes with the original fear memory, it targets the fear memory itself, leading to an abrupt change in avoidance behaviour when confronted with the feared situation the following days [9, 20, 21].

Pharmacological interference with reconsolidation has progressed from animals to humans by—for instance—using the noradrenergic beta-blocker propranolol [10]. While the exact neurobiological processes behind how propranolol alters reconsolidation in humans are not yet fully understood, the beta-blocker is thought to interfere with protein synthesis indirectly by inhibiting the action of noradrenaline-stimulated molecules that are required for memory processes, thereby interfering with the restabilization of the fear memory [24–26]. Human fear-conditioning studies from our research group have repeatedly demonstrated that the affective or defensive component of a fear memory, as assessed with the fear-potentiated startle response, can be abruptly attenuated when propranolol was administered in combination with reactivation of the previously acquired fear memory (i.e., reconsolidation intervention), whereas self-reported threat expectancies initially remained unaffected [8, 14, 27–31]. However, the attenuation of the fear-potentiated startle response was not always replicated [32–34]. A study translating these promising findings to existing fears revealed remarkable results: Soeter and Kindt [9] showed that individuals with sub-clinical spider fear, who received propranolol after a brief memory reactivation procedure involving a 2-min exposure to a tarantula^1^, transformed their fearful avoidance into approach behaviour as assessed with a tarantula and a baby tarantula behavioural approach task (BAT), the latter serving as a generalization stimulus. A reduction in self-reported spider fear followed the behavioural transformation at the 3-month follow-up. In contrast, individuals who received placebo or took propranolol without memory reactivation did not improve. All effects were maintained after one year. Recent findings regarding the translational value of reconsolidation interventions however are either mixed or challenging to interpret due to methodological shortcomings: We found support for a reduction in sub-clinical spider fear after a reconsolidation intervention, but this study did not include a placebo-control condition [13]. Two reconsolidation intervention studies for spider fear and fear of public speaking found a substantial fear reduction following a reconsolidation intervention, but without reliably outperforming placebo [11, 12]. Sample sizes were small and behavioural avoidance was not assessed at baseline, limiting the interpretability of these two studies.

The notable discovery that self-reported spider fear was not initially reduced after the reconsolidation intervention in Soeter and Kindt’s study, and instead followed the behavioural transformation [9], aligns with the striking dissociation reported in human fear-conditioning studies, in which the fear-potentiated startle response was abruptly attenuated, but self-reported threat expectancies initially remained unaffected and decreased over the course of multiple extinction trials [8, 27, 29, 30,2]. Such a dissociation challenges a core principle of CBT, in which updating maladaptive cognitions is often seen as critical for subsequent behavioural changes [9, 21, 35]. The reconsolidation intervention seems to work in the reversed order by abruptly reducing fear behaviour following the initial weakening of the fear memory during the reconsolidation window, after which maladaptive cognitions may change [9, 21]. As a reduction in self-reported spider fear was detected at the 3-month follow-up in Soeter and Kindt’s study but was not measured between 11 days and 3-month post-treatment [9], it is currently unclear when and how these changes in self-reported fear occurred. In sub-clinical reconsolidation intervention studies, participants re-encounter the feared stimulus during their follow-up assessments where they perform BATs to measure avoidance behaviour. This re-encounter may not only serve as a fear assessment but might be a critical addition to the reconsolidation intervention itself to substantiate changes in fear [7, 21]. In clinical fears, dysfunctional beliefs towards the phobic cue are closely linked to one’s identity (e.g., “*I can’t cope with spiders and must run away”*). Testing one’s fear behaviour may offer an opportunity to challenge safety behaviours after treatment and to disconfirm these maladaptive beliefs [36]. As such, re-encountering the phobic cue after the reconsolidation intervention may help to update one’s autobiographical memory and how one relates to the feared situation (e.g., *“I am able to deal with spiders when I see one”)*, and this re-encounter may therefore constitute a critical addition to the reconsolidation intervention’s treatment protocol.

**Fig. 1:**
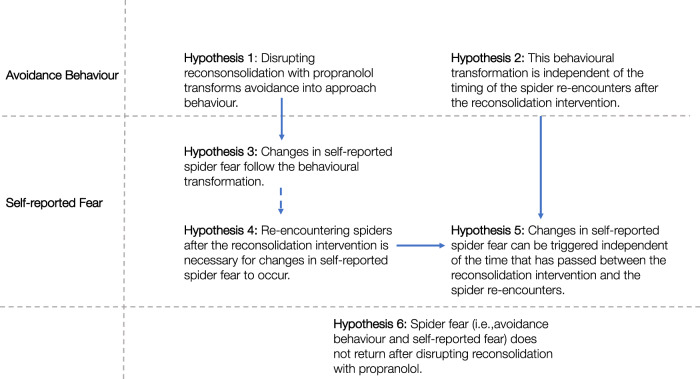
Illustration of hypotheses. We expected that participants who received propranolol after the memory reactivation procedure would approach a tarantula and a house spider more when they returned to the laboratory two days after treatment than those who received placebo (Hypothesis 1). If the reconsolidation intervention neutralizes the affective value of a fear memory as some previous research suggests, avoidance behaviour towards the feared situation might be reduced independent of how much time has passed since treatment. Thus, we tested whether individuals who received propranolol after memory reactivation and returned to the laboratory four weeks post-treatment would show more approach behaviour compared to those who received placebo (Hypothesis 2). In line with previous research, we expected that changes in self-reported spider fear follow the behavioural transformation (Hypothesis 3). Re-encountering a spider after the reconsolidation intervention may be necessary to trigger changes in self-reported spider fear. Thus, we expected that individuals who received propranolol and returned for their post-test two days after treatment would show a larger decrease in self-reported spider fear within the first four weeks after treatment than those who took propranolol and did not re-encounter spiders in the laboratory until four weeks later (Hypothesis 4). If the reconsolidation intervention fundamentally changes the affective value of a fear memory, then theoretically, the window to trigger a reduction in self-reported fear may not be time limited. Thus, we tested whether changes in self-reported spider fear can be triggered independent of the time that has passed between treatment and re-encountering spiders (Hypothesis 5). Lastly, we tested whether potential treatment effects are maintained at the 3-month and 1-year follow-up (Hypothesis 6).

Shedding light on the dissociation between avoidance behaviour and changes in self-reported fear after a reconsolidation intervention would help to better understand the mechanism of change and to advance current interventions. Thus, we aimed to (1) conceptually replicate Soeter and Kindt’s [9] findings regarding the effectiveness of a reconsolidation intervention for spider fear, extending it to individuals who meet the diagnostic criteria for spider phobia, assessed with a Structured Clinical Interview for DSM-5 disorders (SCID-5-RV). The dissociation in fear expression, in which changes in self-reported fear follow behavioural changes seems to be unique to the reconsolidation intervention, but it is currently unclear when and how these changes occur. Hence, we aimed to (2) assess whether changes in self-reported spider fear automatically follow the reconsolidation intervention, or whether re-encountering the feared situation after this initial treatment session is necessary to observe these changes. Third, if re-encountering the feared situation was necessary to trigger changes in self-reported fear as we hypothesize, then (3) we aimed to test whether the window to re-encounter the phobic cue after the reconsolidation intervention is time limited. If the reconsolidation intervention directly weakens the fear memory as some previous fear-conditioning studies suggested [8–10, 15], then theoretically, a subjective fear reduction may be triggered any time after treatment. See Fig. 1 for our hypotheses.

In this double-blind pre-registered study, 69 individuals with spider phobia underwent a brief memory reactivation procedure with a tarantula that aimed to reactivate and thus destabilize the fear memory, followed by propranolol (i.e., reconsolidation intervention; *Reconsolidation*) or placebo (*Placebo)*^3^. Two groups (Reconsolidation and Placebo) re-encountered spiders two days after treatment in the form of BATs, whereas a third group also received the reconsolidation intervention (i.e., memory reactivation followed by propranolol), but returned for their post-test during which they were subjected to the BATs four weeks after treatment (*Reconsolidation-lateBAT*) to test whether re-encountering the phobic cue within days after treatment is necessary to observe a fear reduction. Changes in avoidance behaviour and self-reported spider fear were monitored for three months with BATs and the Spider Phobia Questionnaire (SPQ) [37] respectively, and again after one year.

## Methods and materials

### Pre-registration

Study procedures, exclusion criteria, and analyses were pre-registered on the Open Science Framework (OSF; osf.io/rj5nf/). Minor changes (see Transparent-Changes-from-Pre-registration supplement) were implemented after data collection started, primarily to ensure feasibility of data collection due to the COVID-19 pandemic and feasibility of statistical analyses.

### Participants

We included 69 individuals (61 female, 7 male, 1 non-binary; *M*_Age_ = 26.78, *SD* = 6.94, range = 18–55) who met the diagnostic criteria for spider phobia based on the SCID-5-RV. Eligible participants had no contraindications for taking propranolol, scored at least 18 on the SPQ, were unable to touch a house spider at intake, and completed the memory reactivation procedure during the treatment session (see S1 for eligibility criteria, S2 for recruitment and screening, S3 for the exclusions and flow chart).

### Ethics approval and consent to participate

This study was approved by the ethical review board of the University of Amsterdam (ID: 2019-CP-11229). Participants provided informed consent and were reimbursed with 75 EUR for completing the 3-month study and another 15 EUR if they returned for the 1-year follow-up. All methods were performed in accordance with the relevant guidelines and regulations.

### Measures and materials

#### Spider Phobia Questionnaire (SPQ; range 0–31) [37]

The 31-item SPQ assessed self-reported spider fear. Participants were required to have an SPQ score of at least 18 at pre-screening and baseline assessment.

#### Patient Health Questionnaire-9 (PHQ-9; range 0–27) [38]

Participants were excluded at pre-screening if they scored at least 15 on the PHQ-9, indicating moderately severe depression [38]. We administered the PHQ-9 at baseline to test for group differences in depressive symptoms.

#### Confidence to touch a tarantula (range 0–100)

This single item measure was used at pre-screening to exclude individuals who scored above 40, possibly indicating insufficient fear towards the treatment stimulus.

#### Tarantula and house spider behavioural approach tasks (TBAT and HBAT)

The BATs were used to assess spider approach behaviour in a stepwise manner at baseline (HBAT), post-test (TBAT, HBAT), 3-month follow-up (HBAT), and 1-year follow-up (HBAT, TBAT). At post-test (either ~2 days or ~4 weeks after the treatment session depending on group), the BATs also served as spider re-encounters and thus as an independent variable to manipulate the temporal distance between treatment and re-encountering spiders. The BATs were modified from Soeter and Kindt to assess a greater range of approach-avoidance behaviour [9]. Participants were free to stop the BAT at any time and the experimenter ended the task if participants did not complete a step within three minutes. In the TBAT (adult female *grammostola porteri*, ~10 cm) participants were asked to approach a tarantula in a terrarium on a table. Steps ranged from standing in front of the terrarium, to touching the tarantula, to placing one hand on the ground of the terrarium with closed eyes while the tarantula was being sprayed with water. In the nine-step HBAT (*eratigena atrica*, ~3 cm, used as a generalization stimulus) steps ranged from sitting in front of a glass jar with the spider inside to letting the spider walk over one’s hands. At each step, participants reported their level of distress (0–100; see subjective units of distress). Participants were excluded at baseline if they touched the house spider (step 7; *n* = 14). See S4 for all BAT steps.

#### Anxiety Sensitivity Index (ASI; range 0–64) [39]

The ASI was measured at baseline to test for group differences in anxiety sensitivity.

#### Treatment credibility ratings (range 0–100)

We asked how much faith participants had in standard exposure therapy and the experimental treatment to explore group differences at baseline.

#### State-Trait Anxiety Inventory (STAI; range 20–80) [40]

The STAI-T (Dutch back-translation) was measured at baseline to test for group differences in trait anxiety. The STAI-S (range 20–80) was measured before and 90 min after memory reactivation to assess whether propranolol had a general fear-dampening effect compared to placebo.

#### Subjective units of distress (SUDS; range 0–100) [41]

We used SUDS to assess self-reported distress throughout the study, including BATs and memory reactivation.

#### Memory reactivation procedure

Memory reactivation was a 1 to 8-min-long spider exposure (*Median [IQR]* = 166 s [138–255 s]) during which participants were asked to enter a separate room with a tarantula in a terrarium on the table, stand on a line 50 cm in front of the terrarium, watch the researcher open the terrarium, place their hands on a glass box directly in front of the open terrarium at the height of the entrance, and keep their hands on the glass box while the tarantula was being sprayed with water twice, causing it to move. If participants were unable to complete memory reactivation within eight minutes (*n* = 8), stopped it due to high distress (*n* = 6), or reported a distress level of less than 50 at all four SUDS that were taken during memory reactivation (*n* = 2), they were excluded from the study. The memory reactivation procedure took place during the treatment session for all groups, and was either followed by a pill of propranolol or placebo, depending on condition.

#### Propranolol and placebo

Within five minutes after memory reactivation, participants either received double-blind a 40 mg pill of propranolol or an identical-looking placebo pill (sugar pill) as in Soeter and Kindt [9]. Accord Healthcare LtD. (UK) produced the propranolol pills, and Huygens Apothecary (NL) provided all pills. See S5 for adherence to treatment instructions before and after the treatment session to ensure the safe intake of propranolol and accuracy of physiological measures.

#### Physiological measures

Blood pressure (BP) and heart rate (HR) were measured at baseline and before memory reactivation to screen for contraindications for propranolol. BP, HR, and salivary alpha amylase (sAA; S6) were measured at the beginning and at the end of the treatment session to monitor whether propranolol physiologically took effect.

See S7 for other exploratory variables.

### Procedure

**Fig. 2:**
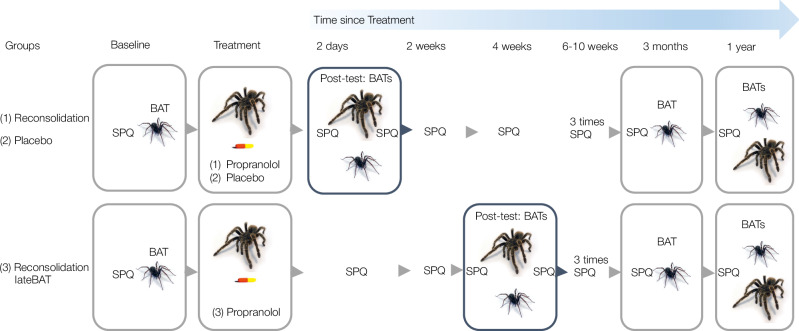
Key aspects of the experimental design. Participants were randomized into one of three groups and the study was double-blind. **Baseline assessment**—The Spider Phobia Questionnaire (SPQ) was followed by a house spider behavioural approach task (HBAT) at intake. **Treatment**—The researcher explained the treatment mechanism and conducted a short spider fear interview after which the memory reactivation procedure with a tarantula took place in a separate room. Memory reactivation was followed by a pill of propranolol (i.e., reconsolidation intervention; *Reconsolidation*) or placebo (*Placebo*) within 5 min. **Self-reported spider fear (SPQ)** was monitored every other week in the three months following the treatment (online when there was no in-person session scheduled). **Post-test** was ~2 days after treatment for the Reconsolidation and the Placebo group, and ~4 weeks after treatment for the *Reconsolidation-lateBAT* group to test whether re-encountering spiders within days after the reconsolidation intervention is necessary to trigger changes in self-reported fear. The post-test involved the SPQ, a tarantula behavioural approach task (TBAT), the HBAT, and another SPQ. The BATs at post-test also served as spider re-encounters to manipulate the temporal distance between treatment and re-encounters. **Follow-ups** took place 3 months and 1 year after treatment, including the SPQ, HBAT, and at 1-year also the TBAT.

Participants were randomized into one of three conditions: (1) Reconsolidation and (2) Placebo, in which participants returned for their post-test involving the spider re-encounters in the form of the BATs two days after treatment, and (3) Reconsolidation-lateBAT, where the post-test including the BATs took place four weeks after the treatment session (see Fig. 2 for the experimental design). The ‘Reconsolidation’ group labels refer to participants undergoing a memory reactivation procedure with a tarantula, followed immediately by propranolol, and thus reflect both the procedure and the hypothesized mechanism of change. Participants had five in-person sessions, consisting of the baseline assessment, the treatment session (i.e., memory reactivation procedure followed by an immediate intake of either propranolol or placebo), the post-test (i.e., spider re-encounters in the form of behavioural approach tasks either ~2 days or ~4 weeks after the treatment session depending on condition), the 3-month follow-up, and the 1-year follow-up. Participants were asked to fill out brief online questionnaires monitoring their spider fear every other week until the 3-month follow-up. Fifty-one participants returned in-person for their 1-year follow-up and another eight participants filled out the online follow-up including the SPQ (*n* = 59), leading to 85% sample retention. Research assistants, blind to condition, conducted the baseline assessment, post-test, and follow-ups. JP was trained by MK and conducted all treatment sessions and remained blind until all measures at treatment were complete. See S8 for session details and detailed experimental procedure, and S9 for randomization and blinding.

### Data analysis

We conducted Bayesian analyses in JASP [42] (jasp-stats.org) and report Bayes Factors (BFs) for inference (S10). BF_Null_ > 1 indicates evidence for the null model compared to all other models. Unless the inclusion of time is indicated, null models control for the random effect of time in our mixed models. BF_10_ > 1 indicates evidence for the alternative hypothesis, e.g., evidence for group differences in t-tests. BF_Inclusion_Interaction_ > 1 indicates evidence for the model including an interaction effect compared to all other models (i.e., null and main). We used default priors for *t*-tests [43, 44], specifically the default Cauchy prior distribution with scale parameter set to $1/\sqrt 2$ unless pre-registered and indicated otherwise, and default multivariate Cauchy prior distributions [45] for repeated-measures ANOVAs (RM-ANOVAs) with scale parameter set to 0.5 for fixed effects and 1 for random effects, as pre-registered. We assessed the robustness of BFs by running our main outcome analyses with two additional priors (S13 and 14). If we identified meaningful group differences at baseline on the HBAT or SPQ, then we additionally ran confirmatory analyses with the baseline assessments as a covariate to check whether the pattern of the results remained similar. Please note that tarantula approach behaviour was not measured at baseline to avoid encountering the treatment stimulus prior to the treatment session, and as such house spider approach behaviour is the only estimate of avoidance behaviour at baseline. Deviating from the pre-registration, Bayesian ordinal regressions using brms [46] in R were omitted because these analyses were underpowered and the pre-registered normal priors for the Bayesian ordinal regressions were too wide and did not provide realistic estimates of the variance in the data (S10). For transparency, ordinal regressions on the main outcome variables are in the supplement (S15). Additionally, we conducted exploratory frequentist RM-ANOVAs on the main outcome variables for increased comparability, for which results were in line with the reported Bayesian RM-ANOVAs (S15).

## Results

### Missing data

From intake until the 3-month follow-up, 2.53% stepwise completion of BATs and 0.18% of SPQ data were missing (S11). For manipulation checks, 14.49% of sAA values and 1.81% of SUDS during memory reactivation were missing. We accounted for missing data in our confirmatory analyses and manipulation checks using multiple imputation by calculating missing values with the average value of 50 imputed data sets using the “mice” [47] package in R. As multiple imputation methods are not recommended for ordinal data [48, 49], we used complete cases for our behavioural data. Results with imputed data until the 3-month follow-up can be found in this supplement (S13 and 14). We also used complete cases for analyses including the 1-year follow-up due to the varying time intervals between data points.

### Manipulation checks

First, RM-ANOVAs found that resting HR, systolic BP, and sAA levels dropped strongly in all groups from before memory reactivation to 90 min after pill intake (BF_Inclusion_Time_ ≥ 953.04), but against our predictions there was no evidence that this decrease was larger after taking propranolol compared to placebo (all BF_Inclusion_Interaction_ ≤ 0.28), see S12 for descriptives and supplementary information regarding adherence to pre-treatment instructions. Second, as predicted, there was no evidence for a group-by-time interaction for state anxiety before and 90 min after the pill intake (BF_Inclusion_Interaction_ = 0.48), ruling out a general fear-dampening effect of propranolol as an explanation for potential treatment effects. Third, RM-ANOVAs indicated no group differences in SUDS during memory reactivation (BF_Null_ = 6.22), suggesting equivalence regarding the experience of subjective distress during memory reactivation between groups. Fourth, we explored whether the duration of the memory reactivation procedure differed between the Reconsolidation (*M* = 168.33 s, range = 107–447 s), Placebo (*M* = 228.64 s, range = 118–499 s), and Reconsolidation-lateBAT (*M* = 233.23 s, range = 115–479 s) groups. An ANOVA indicated no convincing evidence that the groups meaningfully differed in their memory reactivation time (BF_Inclusion_Main_ = 1.07).

### Baseline characteristics

The groups did not differ in self-reported spider fear (SPQ), depression symptoms (PHQ-9), trait anxiety (STAI-T), anxiety sensitivity (ASI), or faith in the experimental treatment (all BF_Null_ ≥ 1) at baseline. We found anecdotal evidence for group differences regarding HBAT stepwise completion at baseline (BF_Null_ = 0.61) and age (BF_Null_ = 0.77). Although these inconclusive data may not indicate a meaningful difference in HBAT approach behaviour at baseline, we checked whether the pattern of the results remained similar when adding baseline HBAT approach behaviour as a covariate in our confirmatory analyses. Lastly, we found moderate evidence for group differences regarding the credibility of standard exposure treatment (BF_Null_ = 0.19), but not for credibility of the experimental treatment (BF_Null_ = 4.63). See Table 1 for baseline descriptives.

**Table 1:** Mean scores and standard deviations (SDs) by group for baseline measures.

*Baseline measures*	Reconsolidation (*n* = 22)		Placebo (*n* = 25)		Reconsolidation-lateBAT (*n* = 22)		
	*Mean*	*SD*	*Mean*	*SD*	*Mean*	*SD*	BF_Null_
*Spider Phobia Questionnaire*	23.27	2.71	22.96	2.72	22.27	3.18	4.85
*House spider BAT step*	3.59	1.74	2.60	2.33	4.05	1.62	0.61
*Patient Health Questionnaire-9*	2.82	3.53	3.00	2.02	2.50	1.79	6.90
*Trait anxiety*	33.59	9.54	37.92	7.78	36.36	6.84	2.29
*Anxiety Sensitivity Index*	15.77	10.25	12.20	6.61	12.41	5.51	2.58
*Treatment credibility – Exposure*	58.09	21.96	65.52	18.75	46.46	21.23	0.19
*Treatment credibility – Experimental*	65.50	18.59	66.72	14.06	60.59	20.60	4.63
*Age (years)*	29.68	9.71	26.04	5.10	24.77	4.25	0.77

Bayes Factor (BF)_Null_ > 1 indicates that the data are more likely under the null model compared to the model including the main effect, e.g., for the Spider Phobia Questionnaire, the data are 4.85 times more likely under the null model than under the model including the main effect.

### Avoidance behaviour

#### Primary behavioural outcome: Tarantula approach behaviour

**Fig. 3:**
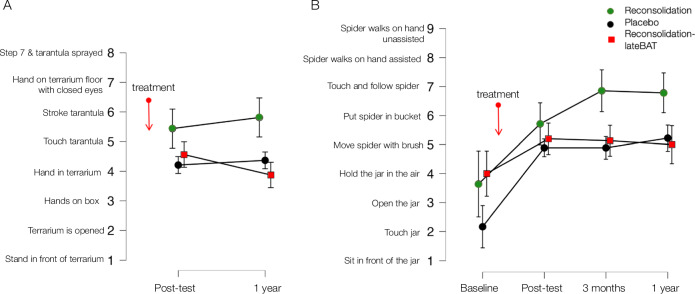
Approach behaviour: Tarantula and house spider behavioural approach tasks. Error bars show 95% credible intervals. **A** Steps completed in the tarantula behavioural approach task (TBAT), measured at post-test (~2 days after treatment for the Reconsolidation and Placebo groups and ~4 weeks after treatment for the Reconsolidation-lateBAT group) and at the 1-year follow-up. Higher stepwise completion indicates more approach behaviour. *N* = 51. **B** Steps completed in the house spider behavioural approach task (HBAT), measured at baseline, post-test, 3-month follow-up, and at the 1-year follow-up. *N* = 47.

A one-tailed Mann–Whitney *U* test indicated anecdotal but inconclusive evidence that participants who received propranolol approached a tarantula more two days after treatment (Reconsolidation) compared to those who received placebo (BF_10_ = 1.53)^4^ (hypothesis 1). The evidence for more approach behaviour in the Reconsolidation-lateBAT group compared to Placebo at their respective post-tests was negligible (BF_10_ = 1.08) (hypothesis 2). A RM-ANOVA (BF_Inclusion_Main_ = 7.60) indicated that participants in the Reconsolidation group approached the tarantula most over the course of one year compared to both, Placebo (BF_10_ = 34.77) and Reconsolidation-lateBAT (BF_10_ = 52.46), whereas Placebo and Reconsolidation-lateBAT did not differ (BF_10_ = 0.25) (see Fig. 3a). The pattern of these results remained similar when including HBAT steps at baseline as a covariate (S13).

#### Secondary behavioural outcome: House spider approach behaviour

Against our predictions, two one-tailed Mann–Whitney *U* tests indicated no evidence for a larger increase in house spider approach behaviour from baseline to post-test between either Reconsolidation and Placebo (BF_10_ = 0.16), or Reconsolidation-lateBAT and Placebo (BF_10_ = 0.12) (hypothesis 1 and 2). However, two RM-ANOVAs including all three groups suggested a group-by-time interaction from baseline until three months (BF_Inclusion_Interaction_ = 14.48) and one year (BF_Inclusion_Interaction_ = 18.74) after treatment (see Fig. 3b). To further examine long-term differences in house spider approach behaviour between the Reconsolidation and the Placebo group, we tested their group-by-time interaction from post-test until the 1-year follow-up. At post-test these two groups did not differ in house spider approach behaviour (BF_10_ = 0.33), whereas there was anecdotal evidence that they differed at baseline (see baseline characteristics). There was anecdotal evidence for the model including a group-by-time interaction (BF_Inclusion_Interaction_ = 1.96), also when including house spider approach behaviour at baseline as a covariate (BF_Inclusion_Interaction_ = 1.44). Comparisons of RM-ANOVAs indicated that the Reconsolidation group approached the house spider more from post-test until 1-year after treatment compared to Placebo (BF_10_ = 38.74). Regarding long-term effects at the 3-month and 1-year follow-up, a RM-ANOVA confirmed more house spider approach behaviour in the Reconsolidation compared to the Placebo group (BF_10_ = 15.73; hypothesis 6), as the data were most likely under the model including a main effect of group (BF_Inclusion_Main_ = 1.47), also when including HBAT steps at baseline as a covariate (BF_Inclusion_Main_ = 3.11, BF_10_ = 36.46). Against our predictions, there was no evidence that Reconsolidation-lateBAT and Placebo differed at the 3-month and 1-year follow-up as indicated by a RM-ANOVA (BF_Null_ = 2.33; hypothesis 6).

### Self-reported spider fear

**Fig. 4:**
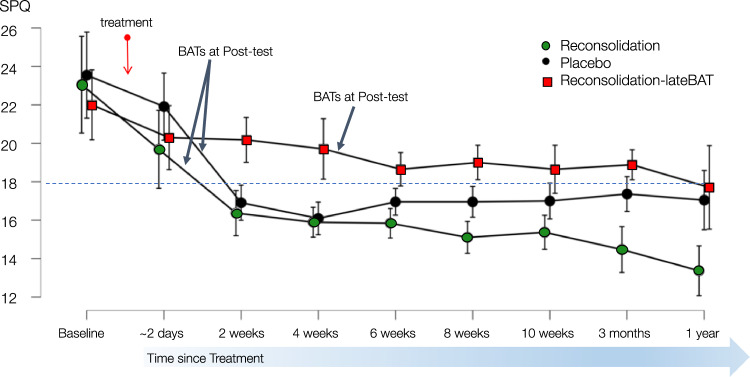
Self-reported spider fear over one year. SPQ: Spider Phobia Questionnaire (0–31). Error bars show 95% credible intervals. Baseline: Pre-treatment. Treatment involved a memory reactivation procedure with a tarantula, followed by a pill of propranolol (i.e., reconsolidation intervention; *Reconsolidation*) or placebo (*Placebo*). Post-test involved the spider re-encounters in the form of behavioural approach tasks (BATs), which took place ~2 days after treatment for Reconsolidation and Placebo, and ~4 weeks after treatment for *Reconsolidation-lateBAT*. *N* = 59.

We first explored global changes in self-reported spider fear over the course of one year. An overarching RM-ANOVA with all three groups from baseline to 1-year post-treatment suggested a strong group-by-time interaction (BF_Inclusion_Interaction_ = 254.99, see Fig. 4) that remained meaningful when controlling for HBAT steps at baseline as a covariate (BF_Inclusion_Interaction_ = 250.66). Post-hoc tests indicated anecdotal evidence that SPQ scores in the Reconsolidation group were lower compared to Placebo (BF_10_ = 2.25) over the course of one year. A RM-ANOVA from four weeks post-treatment until the 1-year follow-up indicated a strong group-by-time interaction (BF_Inclusion_Interaction_ = 78.81) between Placebo and Reconsolidation, suggesting that the active treatment is more effective in the long term. In contrast, there was strong evidence that Reconsolidation-lateBAT reported more spider fear over one year compared to Reconsolidation (BF_10_ = 777.24), whereas Placebo and Reconsolidation-lateBAT did not seem to differ over one year overall (BF_10_ = 0.82).

Next, we zoomed in on whether changes in self-reported spider fear followed the behavioural changes (hypothesis 3). As predicted, no group differences in the SPQ were found two days after treatment, before any of the groups had re-encountered spiders in the form of BATs (BF_Null_ = 3.44). Despite a strong decrease in self-reported spider fear following the spider re-encounter two days after treatment, we did not find the predicted larger decrease in SPQ scores in the Reconsolidation compared to the Placebo group from 2 days after treatment until the 3-month follow-up (BF_Null_ = 3.29) as both groups initially showed a strong decrease in self-reported spider fear in this time period. As such, long-term benefits of Reconsolidation compared to Placebo seem to emerge later than predicted.

With regards to hypothesis 4, a RM-ANOVA suggested a group-by-time interaction between the Reconsolidation and the Reconsolidation-lateBAT group from 2 days to 4 weeks after treatment before the Reconsolidation-lateBAT group re-encountered spiders (BF_Inclusion_Interaction_ = 4.50) suggesting that re-encountering spiders after treatment is indeed necessary to trigger a decrease in self-reported spider fear (hypothesis 4).

Lastly, a pre-registered one-tailed *t*-test comparing SPQ scores between the two reconsolidation groups two months after their respective spider re-encounters (i.e., eight weeks after treatment for the Reconsolidation group and three months after treatment for the Reconsolidation-lateBAT group) yielded anecdotal evidence for more spider fear in the Reconsolidation-lateBAT group (BF_10_ = 1.88). Against our predictions, the Reconsolidation-lateBAT group also reported higher SPQ scores compared to Placebo in the two months after the delayed spider re-encounter, i.e., four to twelve weeks post-treatment (BF_Inclusion_Interaction_ = 3.71). Together with the previously discussed global changes in self-reported fear over the course of one year, these findings suggest that changes in self-reported spider fear *cannot* be triggered independent of the time that has passed between the treatment and the spider re-encounters (disconfirming hypothesis 5).

Overall, these findings indicate that changes in self-reported spider fear follow the behavioural change (but also in the Placebo group) and are triggered by spider re-encounters, which should take place *within days* after the reconsolidation intervention.

## Discussion

Our findings do not confirm that a reconsolidation intervention is more effective in reducing spider avoidance behaviour (hypothesis 1) and self-reported fear than a placebo intervention, at least in the short term. In the long term, the reconsolidation intervention was more effective than placebo, but only when the phobic stimulus was re-encountered within days after treatment (disconfirming hypothesis 2). Specifically, we found less tarantula avoidance behaviour and self-reported fear over the course of one year when spiders were re-encountered two days after the reconsolidation intervention, but not when the behavioural test was conducted four weeks after the intervention. Memory reactivation followed by placebo reduced avoidance behaviour and self-reported spider fear as well, but less so long-term.

Two previous reconsolidation intervention studies from our research group also found a substantial fear reduction after treatment, but where propranolol did not reliably outperform placebo [11, 12]. Although we observed a similarly strong or even stronger fear reduction after the reconsolidation intervention in the current study compared to Soeter and Kindt, our findings contrast with Soeter and Kindt’s study, in which control groups neither showed a reduction in their self-reported fear, nor avoidance behaviour [9]. In the light of the unexpectedly strong fear reduction after placebo in the current study and the two previous studies [11, 12], alternative accounts that may have reduced spider fear need to be considered, such as placebo and exposure effects as these may have resulted in a (temporarily) similarly strong fear reduction in both groups [11, 12]. Regarding placebo effects, Siegel and Peterson [50] showed that placebo pills can increase self-efficacy: Spider fearful individuals who falsely believed that they took propranolol approached a tarantula more compared to individuals who knew that they took placebo. Regarding exposure effects, older and stronger memories are less susceptible to reconsolidation interference [7, 21, 24, 51] and require longer memory reactivation procedures to enter a labile state than laboratory-induced memories [51, 52]. Given that the current study focused on a clinical rather than sub-clinical sample, we developed a more challenging memory reactivation procedure compared to Soeter and Kindt [9]. This may have unintentionally resulted in exposure effects (through the formation of an inhibitory extinction memory), at least for some individuals in our study [1, 21]. The spider re-encounters in the form of the tarantula and house spider BATs two days after treatment may then have strengthened extinction learning [1, 21]. Here, the tarantula and house spider BATs may have served as added exposure or as an extinction reminder.

Still, the overall pattern of the results is difficult to reconcile with placebo or exposure effects alone. The anticipated stronger fear reduction after undergoing memory reactivation followed by propranolol, as compared to placebo, manifested later than hypothesized. This delay was not attributable to insufficient fear reduction in the reconsolidation intervention group, but rather to the unforeseen significant reduction in fear observed in the placebo group. Hence, we cannot rule out the possibility that the observed reduction in fear resulted from positive treatment expectations (i.e., placebo effect) bolstered by extinction learning. Yet, the observed long-term advantage after the reconsolidation intervention may align more closely with the reconsolidation hypothesis than with the expectancy/extinction hypothesis, at least for a subset of the active treatment group. Specifically, the reconsolidation intervention group benefited more from the brief exposure during the post-test, as only this group showed an advantage in self-reported fear and fear behaviour long-term. In contrast, the initial improvement in self-reported fear and approach behaviour observed in the placebo-control group persisted without further improvement from post-test to the 1-year follow-up. Reconsolidation interference is thought to directly weaken (the affective value of) the fear memory, thereby potentially preventing a return of fear [7, 10]. In contrast, extinction learning is believed to merely form an inhibitory memory that competes with the original fear memory, possibly hindering further improvements or causing a return of fear [1, 5, 6]. Thus, while both groups benefited from the treatment, it is plausible that the underlying mechanism of change differed between the memory reactivation procedure followed by propranolol versus placebo, resulting in long-term advantages after the reconsolidation interference procedure. However, we can never be certain that propranolol actually interfered with the process of memory reconsolidation, as there is currently no molecular or real-time marker during the reactivation procedure in humans to confirm that the reconsolidation window indeed opened up [21, 53].

If the reconsolidation intervention directly weakens the affective value of the fear memory as some previous studies suggested [8–10, 15], then changes in self-reported fear, or how people relate to the phobic cue, may be triggered any time after the treatment. We tested this hypothesis and found that the feared stimulus needs to be re-encountered within days, rather than weeks, after the treatment session to observe such changes (disconfirming hypothesis 5). Hence, the reconsolidation intervention alone does not seem to be sufficient to change how people think about the phobic cue in real-life situations, challenging the notion that reconsolidation interventions merely require a single treatment session to reduce fear [7, 21]. Instead, confronting the phobic cue within days after the reconsolidation window has already closed seems to be critical to consolidate the initial effects of the reconsolidation intervention. In line with CBT principles, the new experience of being able to approach the phobic cue without panic seems to be necessary to subsequently change one’s self-image as a phobic individual, reflected in a decrease in self-reported fear. This extended CBT-like approach may prove effective by targeting safety behaviours, thereby facilitating the updating of autobiographical memory within days following the initial treatment (e.g., *I didn’t feel the panic I expected, so I may now be able to deal with spiders in my daily life)*. The current findings thus support the multifaceted nature of emotional memory [21, 54–57]. Conceptually, enduring treatment effects in clinical samples appear to require the updating of multiple aspects of fear—affective, behavioral, and cognitive. However, this process may not occur simultaneously and may require different intervention techniques for each facet.

Lastly, it is important to note that re-encountering the phobic cue also resulted in a reduction in self-reported fear in the placebo-control group. This finding emphasizes that it may be premature to celebrate the effectiveness of the reconsolidation intervention alone to treat anxiety and related disorders. Further research is needed to shed light on the mechanisms of change underlying exposure- versus reconsolidation-based interventions for clinical fears. While fear-conditioning studies in both rodents and humans have demonstrated that extinction learning and reconsolidation interference reduce fear through different mechanisms [21, 29, 51, 58], long-lasting fears and phobias are more complex and resistant to change than laboratory-induced fear (memories), and so may be the mechanisms to change them [21, 24]. A translational approach can help to understand this complexity, but several aspects of fear-conditioning studies in the laboratory cannot be seamlessly applied to the treatment of phobias and other anxiety disorders. For instance, fear-conditioning studies have shown that reconsolidation is influenced by various factors such as learning history, age, and strength of the targeted memory [21, 24, 51]. In the laboratory setting, researchers can manipulate these variables by adjusting the conditioning or reactivation procedures, which is not feasible when working with people with long-lasting fears and phobias. As we do not have an independent marker to indicate whether reconsolidation is triggered [21, 53], we can often only infer the potential mechanisms of change indirectly based on how and when different aspects of fear memory expression (e.g., avoidance behaviour and self-reported fear) change over the course of treatment.

### Limitations

This placebo-controlled study systematically shed light on the dissociation between avoidance behaviour and self-reported fear after a reconsolidation intervention, but there are some limitations to consider. First, while we found a strong decrease in salivary alpha amylase levels, systolic blood pressure, and heart rate from before memory reactivation to 90-min after pill intake, we did not find the predicted stronger decrease in these measures after taking propranolol compared to placebo as was observed in Soeter and Kindt [9]. This finding is not unique to the current study [12], but means that we cannot prove that propranolol physically took effect. The strong reduction of physiological measures in all groups may be due to high treatment-related anticipatory distress and the subsequent resting period in this phobic sample, which may have concealed detectable differences in arousal due to propranolol versus placebo.

Second, we found anecdotal evidence for baseline group differences in house spider approach behaviour, which complicates the interpretation of behavioural treatment benefits. However, the three groups neither differed in their self-reported spider fear at baseline, nor two days after treatment before any of the groups were subjected to the BAT. The three groups also did not differ in their house spider approach behaviour at post-test (before group differences re-emerged at the follow-ups). Thus, at the minimum, long-term changes in fear were not entirely driven by a potential baseline difference in avoidance behaviour. Furthermore, when we included house spider approach behaviour at baseline as a covariate in the relevant analyses (see S13 and 14), the pattern of the results remained largely similar.

Third, a full factorial design including an additional placebo-control group with spider re-encounters four weeks after treatment would have been ideal, but for reasons of feasibility we decided against it. As the Reconsolidation-lateBAT and Placebo groups differ in two aspects, i.e., the timing of the spider re-encounters after treatment *and* the type of pill (propranolol versus placebo), we only report comparisons between these two groups (pre-registered) in the context of some valid comparisons between the other groups.

Fourth, differences in self-reported spider fear between our active treatment and placebo-control group were not robust between the full sample and the sample retained one year post-treatment, even though only three participants from both groups did not return. Specifically, the advantage in self-reported fear of the active treatment is only statistically meaningful in the 1-year sample, but not in the full sample, which means that these results need to be interpreted with caution (S16). Nonetheless, the emerging interaction in self-reported fear between the active and placebo-control group was already visible in the full sample towards their 3-month follow-up, suggesting that the direction of these effects is robust.

Fifth, our sample was relatively small due to the clinical nature of this study. Our power analysis was based on Soeter and Kindt [9], but in recent years it has become clear that initial findings often present an overestimation of the true effect size [59]. Although our sample size allowed us to detect substantially smaller effects than those reported by Soeter and Kindt [9], some analyses were still underpowered because group differences between our active treatment and placebo-control group were smaller than expected. See S17 for our power analyses and sample size justification.

## Conclusion

Memory reconsolidation interventions are thought to work through a fundamentally different mechanism than exposure treatment by weakening fear memories directly. Even though we also observed a fear reduction in the placebo-control group, our findings support reconsolidation-inspired interventions as a promising and efficient way to reduce phobia symptoms long-term. However, a brief re-encountering of the feared situation after the initial treatment session is critical to observe a lasting fear reduction and the window of opportunity to trigger changes in self-reported fear seems time limited: Re-encountering the phobic cue two days, but not four weeks, after treatment led to a strong reduction in self-reported spider fear in this study. These findings challenge the idea that a reconsolidation intervention alone is sufficient to treat clinical fears and suggest that multiple facets of the fear memory need to be targeted. Clinically, experiencing the behavioural change *within days* after treatment seems necessary to observe long-lasting treatment effects, which means that testing your fear behaviour after the treatment should be a critical addition to the reconsolidation intervention itself.

## Supplementary information

Supplementary Materials

## Data Availability

Anonymised data for confirmatory and exploratory analyses and supplementary information can be found at the OSF web page for this study (osf.io/rj5nf/). Supplementary information is also available at Molecular Psychiatry’s website.

## Footnotes

1 The memory reactivation procedure is a brief but intense exposure to a tarantula and is designed to trigger reconsolidation, not extinction. Hence, we refer to it as ‘memory reactivation’ and not ‘exposure’ to avoid confusion regarding the mechanism of change. Memory reactivation aims to reactivate the fear memory to destabilize it, after which propranolol is administered to interfere with reconsolidation. This combination is crucial for a reconsolidation intervention [9, 10]. The process of reconsolidation is thought to be complete after a night of sleep [20].

2 Avoidance behaviour and self-reported spider fear are inherently imperfect translations of the fear-potentiated startle response and self-reported threat expectancies, but are more ecologically valid, clinically relevant, and thus suitable for (sub-)clinical research. We chose them as proxies as they are thought to tap into the similar memory systems.

3 In humans, we can only infer whether reconsolidation interference was successful based on self-reported and behavioural results as there is no real-time marker during the reactivation procedure or molecular measure to attest that reconsolidation actually occurred [21, 53]. Thus, although the memory reactivation procedure was developed to open the reconsolidation window, we cannot prove that reconsolidation interference took place, and as such the group labels reflect both the procedure and the hypothesized mechanism of change.

4 For reference, when using a frequentist Mann–Whitney *U* test, this group difference in tarantula approach behaviour between placebo and propranolol two days after treatment is significant, both, one-sided (*U* = 243, *p* = 0.023) and two-sided (*p* = 0.046).
