# Supplementary material for: Re-encountering the phobic cue within days after a reconsolidation intervention is crucial to observe a lasting fear reduction in spider phobia
Source: Mol Psychiatry. 2025 Jan 9;30(6):2729–38. doi: 10.1038/s41380-024-02882-1 (PMC12092263; doi:10.1038/s41380-024-02882-1)
Supplement: Supplementary file 1 — Supplementary Materials [file 41380_2024_2882_MOESM1_ESM.pdf]

Supplementary Materials:

Re-encountering the phobic cue within days after a reconsolidation intervention is crucial to observe a lasting fear reduction in spider phobia

Jacqueline Peters[a], Anna I. Filmer[a], Johnny B. van Doorn[a], Vivian N. Metselaar[a], Renée M. Visser[a], and Merel Kindt[a]

[a] Department of Psychology, University of Amsterdam, Amsterdam, The Netherlands

Methods and Materials

Participants

S1: Full List of Eligibility Criteria

Eligible participants had no contraindications for taking propranolol, met the diagnostic criteria for spider phobia based on the SCID-5 (modified for spider fear), scored at least 18 on the Spider Phobia Questionnaire (SPQ), were unable to touch a house spider at baseline during a behavioural approach task (BAT), and were no more than 40% confident that they could touch a tarantula. Further, participants did not have a current manic, depressive, or psychotic episode, a current diagnosis of generalized anxiety disorder, or a lifetime diagnosis of psychoticism. They had to score less than 15 on the patient health questionnaire-9 (PHQ-9), screening for current depressive symptoms. Participants were also excluded if they had undergone previous therapy for spider fear that included in vivo exposure to a tarantula, were more afraid of house spiders than tarantulas, or more disgusted of spiders than afraid. Lastly, they had to be comfortable with participating in English, could not have participated in any research relating to their fear of spiders that involved spider encounters or behavioural approach tasks, and could not have taken a beta-blocker more than 3 times in the past 2 years and no more than 2 of those in the past year.

The following criteria contraindicated the intake of propranolol, which means individuals who met them were unable to participate, unless a participant's general practitioner signed a form stating that it was safe for them to take propranolol (40mg HCI) in the context of this study.

- Personal history of heart problems, vascular diseases, or irregular heartbeat.
- Personal history of serious (allergic) reactions to propranolol.
- Systolic blood pressure below 100 or a diastolic blood pressure below 60.

- A resting heart rate lower than 60 (or 55 if the participant engages in some sports for 4 hours per week, including cycling; or 50 if the participant engaged in at least 7 hours of sports per week). If the resting heart rate was not lower than 50, but rose to above 60 after light exercise, the participant could still be included.
- Heart rate did not rise from the resting heart rate after two minutes of light exercise (walking up and down two steps repeatedly).
- Current asthma and other lung disorders that were not adequately regulated with medication (but see medication contraindications below).
- Planning to become or currently pregnant or breastfeeding.
- Took any of the following medications (oral or IV): medication that works on the heart, blood pressure lowering agents, ergotamine preparations, insulin, antacids, anti-inflammatory pain killers, antidepressants, antipsychotics, anti-anxiety medication, asthma medication, medication against dizziness, tuberculosis or psoriasis, or any other medicine that interacts with propranolol.

S2: Recruitment and Screening

Participants were recruited with posters, social media advertisements, and the lab recruitment tool of the University of Amsterdam. First, they filled out a pre-screener, assessing eligibility. On the pre-screener, we specified that we were looking for participants whose ‘fear of spiders gets in the way of doing all the things [they’d] like to do, and [who] are motivated to overcome [their] fear’. Participants were informed that the study involved several in-person testing sessions including spider encounters, and that the treatment session required the intake of a pill of propranolol or placebo. If eligible, potential participants were invited to a phone call to conduct the Structured Clinical Interview for DSM-5 disorders (SCID-5-RV) to screen for spider phobia, confirm eligibility, and schedule all sessions.

S3: Exclusions, Dropouts, and Flow Chart

S3a: Exclusions Based on Pre-Registered Criteria

After signing informed consent (N = 126), 48 participants were excluded based on pre-registered criteria. Nine participants' blood pressure (BP) and two participants' heart rate (HR) contraindicated the intake of propranolol. Specifically, Systolic BP < 100, Diastolic BP < 60, and resting HR < 50 were contraindications for propranolol. HR had to rise after two minutes of light exercise to be eligible. Six participants' SPQ scores dropped to below 18 between pre-screening and intake, and 14 participants touched the house spider at intake. Eight participants did not complete the memory reactivation with the tarantula during the treatment session within eight minutes, six participants could not complete the memory reactivation due to high distress, two participants did not experience a distress level of at least 50 (on a 0-100 scale) during the memory reactivation, and one participant experienced more disgust than fear during the memory reactivation. Another five participants were excluded due to COVID-19 related laboratory closures, and four participants dropped out before the three-month follow-up. Thus, after signing informed consent at the intake session, we had to exclude 42.06% of the 126 participants and had a dropout rate of 3.17% until the 3-month follow-up, although only one of the four dropouts occurred after the treatment session.

S3b: Flow Chart

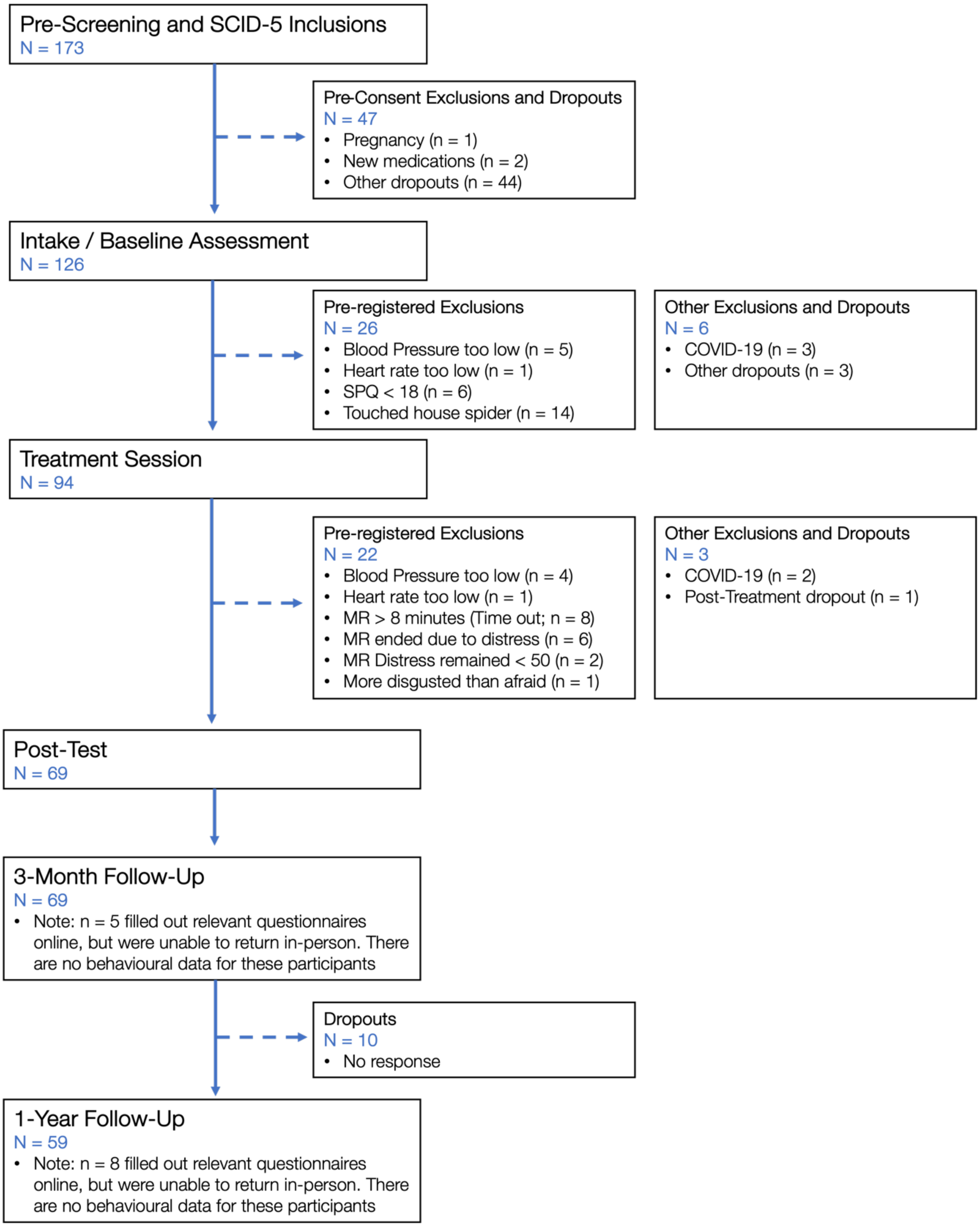

Note. SPQ = Spider Phobia Questionnaire. MR = Memory Reactivation procedure during the treatment session.

Materials and Measures

S4: Steps of the Tarantula (a) and House Spider (b) Behavioural Approach Tasks (BATs)

S4a: Tarantula Behavioural Approach Task

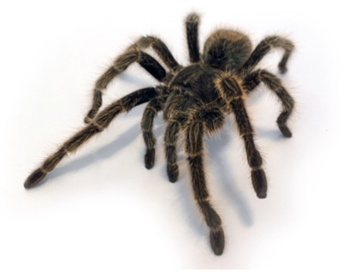

Step	**Description**
1	Stand in front of the terrarium.
2	Watch the experimenter open the terrarium.
3	Place hands on the glass box at the terrarium's entrance.
4	Hover one hand in the centre of the terrarium (through front entrance).
5	Touch the tarantula.
6	Gently stroke tarantula on the back for 5 seconds.
7	Place one hand on terrarium's floor and close eyes for 5 seconds.
8	Place one hand on terrarium's floor and close eyes for 5 seconds while experimenter sprays the spider with water.

Note. Participants were free to discontinue either BAT at their discretion. If attempting any step exceeded three minutes, the experimenter terminated the BAT.

S4b: House Spider Behavioural Approach Task

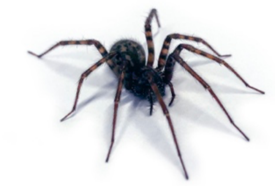

Step	**Description**
1	Sit down in front of the jar with the spider.
2	Put hands on the sides of the jar for 10 seconds.
3	Open the jar.
4	Hold the jar in the air for 10 seconds.
5	Direct spider's movement with a paint brush for 10 seconds.
6	Put the spider from the jar in a bucket.
7	Touch the spider briefly and follow it with one finger for 10 seconds.
8	Let the spider walk on one hand<br>(while the experimenter supports the participant's arm).<br>*Note: This step may be skipped.*
9	Let the spider walk on one hand (without experimenter's support on arm).

Note. Participants were free to discontinue either BAT at their discretion. If attempting any step exceeded three minutes, the experimenter terminated the BAT.

S5: Adherence to Treatment Instructions

For their safety, participants were not allowed to drink alcohol or use drugs in the 24 hours before their treatment session. In the 12 hours before treatment, they were neither allowed to consume caffeine nor engage in intense exercise. To accurately measure salivary alpha amylase, participants were not allowed to eat, smoke, drink anything other than water, or chew gum in the 2 hours before treatment, or brush their teeth 1 hour before treatment. Adherence to these pre-treatment instructions was checked at the beginning of the treatment session, please see the OSF web page to view the adherence form that participants received (osf.io/yczaw). After the treatment session, participants were not allowed to drink alcohol, use drugs, and speak with others in detail about the memory reactivation procedure as this may interfere with memory reconsolidation. Adherence to these rules was checked 2 days after treatment when participants filled in their first post-treatment questionnaire.

S6: Salivary Alpha Amylase (sAA).

To collect sAA, participants placed a saliva sample for three minutes in the centre of their mouth. Saliva samples were stored at < -25 Celsius as soon as possible after collection. Concentration of sAA was assessed by an enzyme kinetic method at the Dresden LabService GmbH (DE). The intra and interassay coefficients for sAA were both below 9%. Of note, the package including all saliva samples was temporarily lost by PostNL during their delivery problems in the winter of 2021/2022, and arrived at its destination with a delay of approximately three weeks. According to the Dresden LabService, this probably did not detrimentally affect sAA levels.

S7: Other Exploratory Variables

During the baseline assessment, we exploratorily asked participants how many years they had been fearful of spiders and at what age their spider fear began. When assessing participants' self-reported spider fear with the SPQ every two weeks, we also monitored encounters with spider stimuli that were not related to this study. In the closing questionnaire of the 3-months follow up, we asked several exploratory questions regarding participants' spider fear and their experience in this study. In the closing questionnaire of the 1-year follow-up, we exploratorily administered the Spider Distress Scale (SDS; Peters et al., 2022), which measures spider fear and disgust, and asked whether participants experience more disgust or fear towards spiders. Lastly, participants were asked several exploratory questions regarding their spider fear and their experience in this study.

Procedure

S8: Session Details and Experimental Design

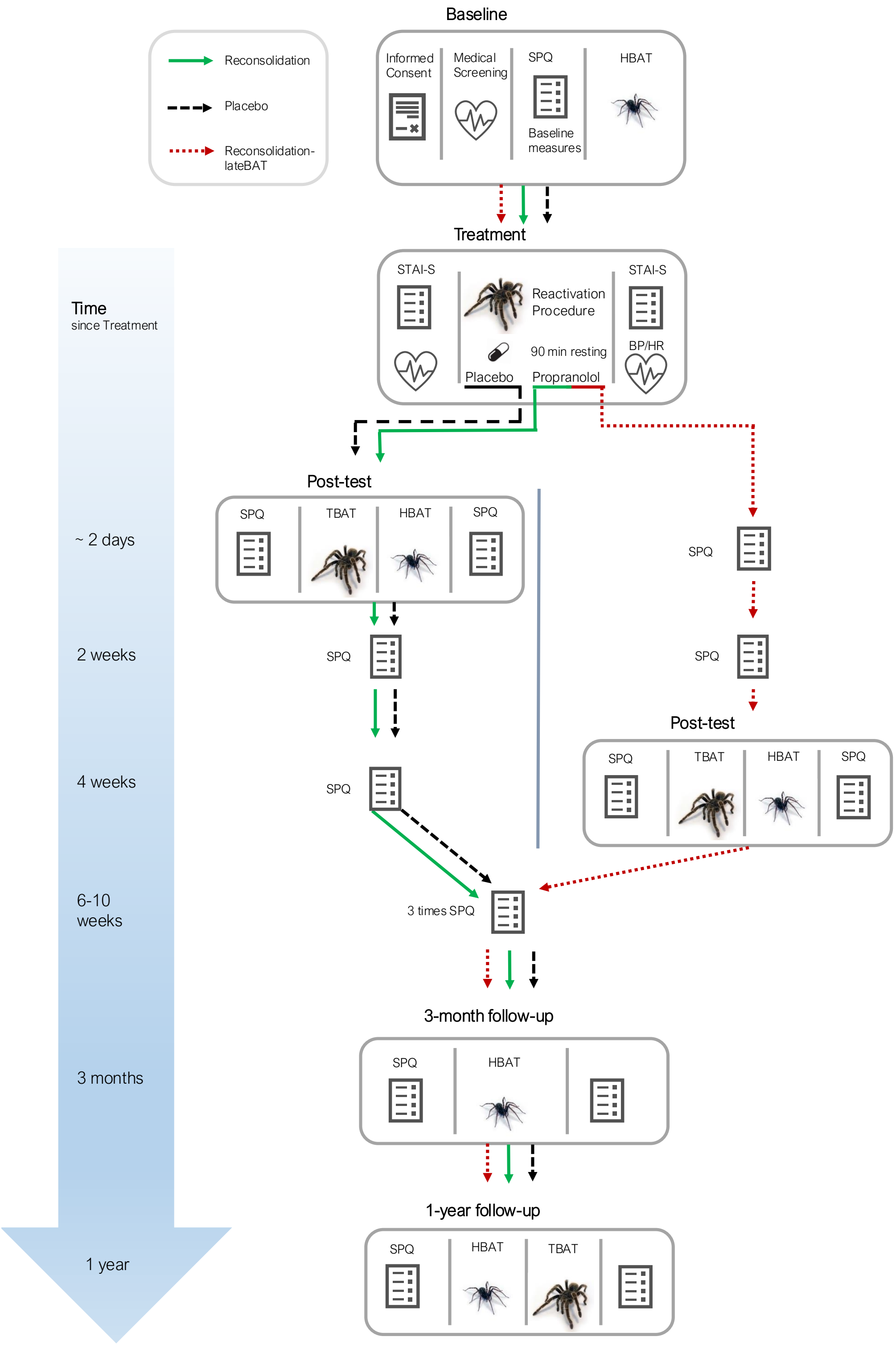

Note. Detailed experimental design. Participants were randomized into one of three groups and the study was double-blind. **Baseline assessment** – Informed consent was followed by a medical screening to check for contraindications for propranolol, including measuring blood pressure (BP) and heart rate (HR) at rest and after

light exercise. Baseline questionnaires followed including the Spider Phobia Questionnaire (SPQ) and lastly participants engaged in a house spider behavioural approach task (HBAT) to measure spider avoidance behaviour. **Treatment** – The State Anxiety Inventory (STAI-S) and a saliva sample were followed by a medical screening including BP and HR, and a treatment adherence check (S5). The researcher then explained the treatment mechanism and conducted a short spider fear interview, after which they went to a separate room for the memory reactivation procedure with a tarantula. Memory reactivation was followed by a pill of propranolol (i.e., reconsolidation intervention; *Reconsolidation*) or placebo (*Placebo*) within 5 minutes. Participants then rested onsite for 90 minutes to ensure their well-being until propranolol levels peaked. Then they filled out the STAI-S again, provided a saliva sample, and HR and BP measurements were taken again. Participants were asked not to deliberately expose themselves to spiders until their post-test. **Self-reported spider fear (SPQ)** and spider encounters were monitored every other week in the three months following the treatment (online in weeks without in-person sessions). **Post-test** was ~2 days after treatment for the Reconsolidation (green, solid line) and Placebo (black, dashed line) groups, and ~4 weeks after treatment for the *Reconsolidation-lateBAT* (red, dotted line) group to test whether re-encountering spiders shortly after the reconsolidation intervention is necessary to trigger changes in self-reported fear. The post-test involved the SPQ upon arrival at the laboratory, a tarantula behavioural approach task (TBAT), the HBAT, and the SPQ, all separated by 10-min breaks to minimize spillover effects. **Follow-ups** took place 3 months and 1 year after treatment, including a SPQ, a HBAT, a closing questionnaire (S7) and at 1-year also the TBAT.

Baseline Assessment (day 1)

Participants arrived at the laboratory, read the information brochure, and provided informed written consent. Then, participants completed a medical screening, which included filling out a form to confirm that there were no contraindications for propranolol as well as a blood pressure (BP) and heart rate (HR) measurement that was repeated after two minutes of light exercise to ensure that participants' HR rose in response to stepping up and down two stairs. Then, participants filled in the SPQ, PHQ-9, STAI-T, ASI, and treatment credibility ratings to test for potential group differences at baseline, after which they engaged in the HBAT where they were excluded if they touched the spider, in line with Soeter and Kindt (2015).

Treatment (Median = 5, Mean [SD] = 8.81 [11.25] days after baseline assessment)

Participants filled out the STAI-S followed by a brief medical screening, including BP and HR measurements, and a saliva sample. The researcher (JP) then outlined the hypothesized treatment mechanism, specifying that the participant will have to approach a spider during the treatment, after which the participant will receive a pill that is (if propranolol) intended to target the connection between spiders and the participant's fear

response, thereby reducing fear. Then, the researcher conducted a brief spider interview prior to the memory reactivation procedure to help the participant to focus on their fear, which may help some people to reactivate their fear memory more readily once confronted with the tarantula. Specifically, the researcher asked how the participant feels when encountering a spider, which spiders they are particularly scared of, places where they find spiders especially scary, as well as their first and worst experiences with their fear of spiders. Participants were asked to rate their distress level for their first and worst experience with their fear of spiders (0-100). Then, the researcher outlined the procedure of the memory reactivation: The participant and researcher will enter a room with a living tarantula in a terrarium on the table, in which the researcher will ask the participant to actively approach the spider while the participant is encouraged not to engage in safety behaviours.

Then, the participant and researcher went to a different room for the memory reactivation procedure. Memory reactivation was a 1 to 8-minute-long (*Median [IQR]* = 166 seconds [138-255 seconds]) spider exposure during which participants were asked to enter a separate room with a tarantula in a terrarium on the table, stand on a line 50 cm in front of the terrarium, watch the researcher open the terrarium, place their hands on a glass box directly in front of the open terrarium at the height of the entrance, and keep their hands on the glass box while the tarantula was being sprayed with water twice, causing it to move. If participants were unable to complete memory reactivation within eight minutes (n = 8), stopped it due to high distress (n = 6), or reported a distress level of less than 50 at all four SUDS that were taken during memory reactivation (n = 2), they were excluded from the study.

After memory reactivation, participants either received a pill of placebo or 40mg propranolol within five minutes, matching the dosage of Soeter and Kindt (2015). Participants were then asked to rest for 90 minutes onsite to ensure their well-being after

taking the pill. During this time, participants were not allowed to use their phones, smartwatches, or computers to avoid distressing cues, but they were allowed to read a book or magazine. At the end of the resting period, participants filled out the STAI-S, had a brief medical screening, including BP and HR measurements, and provided another saliva sample. Then, the researcher unblinded themselves to the participant's schedule and reminded them of their next in-person session and bi-weekly online questionnaires to monitor their spider fear. Participants were also asked not to deliberately re-expose themselves to spiders until their next in-person session to promote that re-encountering spiders takes place in a controlled setting in line with experimental conditions. Lastly, the researcher unblinded herself to the full condition after the participant had left.

Post-Test (2-3 days (Median = 2 , Mean [SD] = 2.02 [0.15]) or ~4 weeks (Median = 30; Mean [SD] = 30.09 [3.95] post-treatment))

Participants filled in the SPQ, followed by a 10-min break to avoid spillover effects from the questionnaire on spider fear to approaching living spiders and vice versa. Then participants engaged in the TBAT, followed by another 10-min break, and the HBAT. After another 10-min break, participants were asked to fill in the SPQ again before leaving the laboratory.

Follow-ups

During the 3-month follow-up *(Median = 82, Mean [SD] = 84 [6.97*] days post-treatment) participants filled in the SPQ, followed by a break and a HBAT, followed by another break and the closing questionnaire (see S7 for other exploratory variables). The 1-year follow-up was similar to the 3-month follow-up, but a TBAT was added after the HBAT and a 10-min break.

S9: Randomization and Blinding

Research assistants, blind to condition, conducted the baseline assessment, post-test, and follow-ups. Baseline and post-test were never conducted by the same researcher to reduce bias with one exception due to a COVID-19 infection of the scheduled experimenter. Participants were initially scheduled for five sessions with a post-test two days and four weeks after treatment. The baseline researcher unblinded themselves after the session was complete, so that they could inform the participant which of the five scheduled in-person sessions would be "moved online". JP was trained by MK and conducted all treatment sessions (and for blinding purposes no baseline sessions, post-tests, or follow-ups), and remained blind until all measures at treatment were complete. Participants were unblinded after the 1-year follow-up was complete.

Data Analysis

S10: Data Analysis

We used Bayesian statistics and report Bayes Factors (BFs) for inference. $BF_{Null} > 1$ indicates evidence for the null model compared to all other models. Unless the inclusion of time is indicated, null models control for the random effect of time in our mixed models. $BF_{10} > 1$ indicates evidence for the alternative hypothesis, e.g., evidence for group differences in t-tests. $BF_{Inclusion\_Interaction} > 1$ indicates evidence for the model including an interaction effect compared to all other models (i.e., null and main). Pre-registered one-sided t-tests are clearly indicated. We used JASP (JASP Team, 2020) for our main analyses, and brms (Bürkner, 2017) (version 2.19) in R (version 4.3) for ordinal regressions, where we used bridge sampling to obtain BFs for model comparisons (Gronau et al., 2020). We used default priors for t-tests (Rouder et al., 2009; van Doorn et al., 2020), specifically the default Cauchy prior distribution with scale parameter set to $\frac{1}{\sqrt{2}}$, unless pre-registered and indicated otherwise. For mixed ANOVAs we used default multivariate Cauchy prior distributions (Rouder et al., 2012) with scale parameter set to 0.5 for fixed effects and 1 for random effects, as pre-registered. Additionally, we assessed the robustness of BFs by running our main outcome analyses with two additional priors (S13-14). If we identified meaningful group differences at baseline on the HBAT or SPQ, then we additionally ran confirmatory analyses with the baseline assessments as a covariate to check whether the pattern of the results remained similar. Please note that tarantula approach behaviour was not measured at baseline to avoid encountering the treatment stimulus prior to the treatment session, and as such house spider approach behaviour is the only estimate of avoidance behaviour at baseline. Deviating from pre-registration, we omitted the Bayesian ordinal regressions, and report them in the supplement for transparency (S15). These analyses were underpowered and the pre-registered normal priors for the Bayesian ordinal regressions were too wide and did not provide realistic

estimates of the variance in the data. We then used the default multivariate Cauchy prior distributions that we had also pre-registered for our mixed ANOVAs to better compare the ordinal regressions to our mixed ANOVAs. However, BFs often remained unstable after adjusting priors, i.e., the algorithm did not converge despite a high number of alterations, as indicated by the convergence diagnostic Rhat (Bürkner, 2017). Lastly, we conducted exploratory frequentist RM-ANOVAs on the main outcome variables for increased comparability, for which results were in line with the reported Bayesian RM-ANOVAs (S15).

Results

Missing Data

S11: Specification of Missing Data

There were 2.53% missing BAT stepwise-completion data and 0.18% missing SPQ data from intake until the 3-months follow-up. Specifically, one HBAT at post-test in the Reconsolidation group and one TBAT at post-test in the Placebo group were invalid due to experimenter protocol violations and five participants were unable to return in-person at the 3-month follow-up but filled out the self-report questionnaires remotely. For self-reported spider fear, one participant from the Reconsolidation-lateBAT group did not fill out their online questionnaire in the second week post-treatment. For the sAA measurements, 14.49% were missing, and 1.81% of SUDS during memory reactivation were missing. We accounted for missing data in our main confirmatory analyses and pre-registered manipulation checks using multiple imputation by calculating missing values with the average value of 50 imputed data sets using the “mice” (Buuren & Groothuis-Oudshoorn, 2011) package in R. We did not impute 1-year follow-up data because the time interval between the original data collection and the follow-up is too large to justify multiple imputation. As multiple imputation methods are not recommended for ordinal data (Chen et al., 2005; Donneau et al., 2015), we used complete cases for our behavioural data. Results with imputed data until the 3-month follow-up can be found in this supplement (S13-14).

Manipulation Checks

S12: Supplementary Information for Manipulation Checks

S12a: Supplementary Descriptives

Mean Values [SD] of the sAA Levels, Systolic Blood Pressure, and Heart Rate Before and 90 Minutes After Pill Intake for the Three Groups

		Pre-Pill	Post-Pill	Reduction %
Reconsolidation	sAA Level	67.50 [66.58]	26.68 [23.21]	60.47
	Systolic BP	117.82 [10.40]	109.27 [12.13]	7.26
	HR	75.77 [13.11]	58.55 [9.44]	22.73
Placebo	sAA Level	64.71 [58.86]	43.63 [30.79]	32.58
	Systolic BP	116.86 [11.60]	111.30 [9.45]	4.76
	HR	79.40 [15.09]	64.60 [11.06]	18.64
Reconsolidation-lateBAT	sAA Level	50.07 [45.46]	30.89 [20.51]	38.31
	Systolic BP	111.61 [9.17]	103.77 [7.15]	7.02
	HR	71.50 [8.13]	54.96 [7.96]	23.13

Note. There were no missing data for systolic blood pressure (BP) and heart rate (HR). For sAA levels, 14.49% were missing. We first checked for extreme outliers to ensure there were no measurement errors during sAA extraction and analysis. We removed the sAA post-pill value of participant P204 due to an unrealistically high sAA value (i.e., extreme outlier). We then calculated missing values with multiple imputation as described in the manuscript.

Whereas 61 participants adhered to the pre-treatment instructions, eight participants deviated from them (see Table 12b). We did not consider any of these deviations substantial for the absorption of propranolol, and thus included all participants. As these deviations may still have impacted physiological measures, we checked for the robustness of our results regarding HR, systolic BP, and sAA by additionally conducting these manipulation checks with the sub-sample of 61 participants who did not violate these rules. Similar to the whole sample, RM-ANOVAs indicated that there was no evidence for a group-by-time interaction

after taking propranolol compared to placebo regarding the decrease of either HR, systolic BP, or sAA (all $BF_{Inclusion\_Interaction} \leq 0.78$).

S12b: Violations of Pre-Treatment Instructions, Before Pill Intake

Condition	PID	Category Violation	Additional Information
Reconsolidation-lateBAT	P164	Caffeine (< 12 hours)	1 cup 8 hours before treatment
Reconsolidation	P299	Food (< 2 hours)	1 hour 50 minutes before treatment
Reconsolidation	P929	Food (< 2 hours)	Sandwich 1 hour 45 min before treatment
Reconsolidation-lateBAT	P113	Food (< 2 hours)	Piece of bread 1 hour before treatment
Reconsolidation-lateBAT	P962	Food (< 2 hours)	Small piece of bread 1.5 hours before treatment
Placebo	P188	Food (< 2 hours)	Sandwich 1 hour before treatment
Placebo	P159	Food (< 2 hours)	One piece of chocolate 30 minutes before treatment
Reconsolidation	P58	Caffeine (< 12 hours)	1 espresso at 7am

*Note***.** PID: Participant ID.

Main Outcome Analyses

The two sections underneath (S13-14) include the results sections that are found in the main manuscript with additional analyses (indicated in *italics*) for our main outcome variables, including robustness analyses with additional prior specifications, and analyses with imputed data (i.e., imputedDat) compared to complete cases where applicable. We never imputed 1-year follow-up data due to the large time interval between the 3-month follow-up and the 1-year follow-up. To assess the robustness of the Bayes Factors (BFs) prior specification, we ran additional analyses with the Cauchy prior scale parameters set to 0.5 and 1.5 for Student's t-tests and Mann-Whitney U tests. For the RM-ANOVAs, we ran additional analyses with Cauchy scale parameters set to 0.4 for fixed effects 0.5 for random effects (i.e., Priors1) as well as scale parameters set to 1 for fixed effects and 1.5 for random effects (i.e., Priors2).

Avoidance Behaviour

S13: Supplementary Analyses for Avoidance Behaviour

Primary Behavioural Outcome: Tarantula Approach Behaviour. A one-tailed Mann-Whitney U test indicated anecdotal but inconclusive evidence that participants who received propranolol approached a tarantula more two days after treatment (Reconsolidation) compared to those who received placebo (BF_{10} =1.53*, $BF_{10imputedDat}$ = 1.70, $BF_{10prior0.5}$ = 1.91, $BF_{10prior1.5}$ =1.02*)[1] (Hypothesis 1). The evidence for more approach behaviour in the Reconsolidation-lateBAT group compared to Placebo at their respective post-tests was negligible (BF_{10} =1.08, *$BF_{10imputedDat}$ = 1.13, $BF_{10prior0.5}$ =1.27, $BF_{10prior1.5}$ = 0.63;* Hypothesis 2). A RM-ANOVA ($BF_{Inclusion\_Main}$ =7.60, *$BF_{Inclusion\_Main\_HBATCovariate}$ = 6.98,*

[1] For reference, when using a frequentist Mann-Whitney U test, this group difference in tarantula approach behaviour between placebo and propranolol two days after treatment is significant, both, one-sided (U = 243, p = 0.023) and two-sided (p = 0.046).

$BF_{Inclusion\_Main\_Priors1} = 7.87$, $BF_{Inclusion\_Main\_Priors2} = 5.21$) indicated that participants in the Reconsolidation group approached the tarantula most over the course of one year compared to both Placebo ($BF_{10} = 34.77$) and Reconsolidation-lateBAT ($BF_{10} = 52.46$), whereas Placebo and Reconsolidation-lateBAT did not differ ($BF_{10} = 0.25$).

Secondary Behavioural Outcome: House Spider Approach Behaviour. Against our predictions, two one-tailed Mann-Whitney U tests indicated no evidence for a larger increase in house spider approach behaviour from baseline to post-test between either Reconsolidation and Placebo ($BF_{10} = 0.16$, $BF_{10ImputedDat} = 0.15$, $BF_{10prior0.5} = 0.22$, $BF_{10prior1.5} = 0.08$), or Reconsolidation-lateBAT and Placebo ($BF_{10} = 0.12$, $BF_{10prior0.5} = 0.16$, $BF_{10prior1.5} = 0.06$) (Hypothesis 1-2). However, two RM-ANOVAs including all three groups suggested a group-by-time interaction from baseline until three months ($BF_{Inclusion\_Interaction} = 14.48$) and one year ($BF_{Inclusion\_Interaction} = 18.74$) after treatment (see Figure 3b). To further examine long-term differences in house spider approach behaviour between the Reconsolidation and the Placebo group, we tested their group-by-time interaction from post-test until the 1-year follow-up. At post-test these two groups did not differ in house spider approach behaviour ($BF_{10} = 0.33$), whereas there was anecdotal evidence that they differed at baseline (see baseline characteristics). There was anecdotal evidence for the model including a group-by-time interaction ($BF_{Inclusion\_Interaction} = 1.96$), also when including house spider approach behaviour at baseline as a covariate ($BF_{Inclusion\_Interaction} = 1.44$). Comparisons of RM-ANOVAs indicated that the Reconsolidation group approached the house spider more from post-test until 1-year after treatment compared to Placebo ($BF_{10} = 38.74$). Regarding long-term effects at the 3-month and 1-year follow-up, a RM-ANOVA confirmed more house spider approach behaviour in the Reconsolidation compared to the Placebo group ($BF_{10} = 15.73$; Hypothesis 6), as the data were most likely under the model including a main effect of group ($BF_{Inclusion\_Main} = 1.47$, $BF_{Inclusion\_Main\ Priors1} = 1.92$, $BF_{Inclusion\_Main\ Priors2} = 2.65$), also

when including HBAT steps at baseline as a covariate ($BF_{Inclusion\_Main} = 3.11$, $BF_{10} = 36.46$). Against our predictions, there was no evidence that Reconsolidation-lateBAT and Placebo differed at the 3-month and 1-year follow-up as indicated by a RM-ANOVA ($BF_{Null} = 2.33$, $BF_{Null\_Priors1} = 1.93$, $BF_{Null\_Priors2} = 4.05$; Hypothesis 6).

Self-Reported Spider Fear

S14: Supplementary Analyses for Changes in Self-Reported Spider Fear

We first explored global changes in self-reported spider fear over the course of one year. An overarching RM-ANOVA with all three groups from baseline to 1-year post-treatment suggested a strong group-by-time interaction ($BF_{Inclusion\_Interaction} = 254.99$, $BF_{Inclusion\_Interaction\_Priors1} = 314.06$, $BF_{Inclusion\_Interaction\_Priors2} = 31.58$) that remained meaningful when controlling for HBAT steps at baseline as a covariate ($BF_{Inclusion\_Interaction} = 250.66$). Post-hoc tests indicated anecdotal evidence that SPQ scores in the Reconsolidation group were lower compared to Placebo ($BF_{10} = 2.25$) over the course of one year. A RM-ANOVA from four weeks post-treatment until the 1-year follow-up indicated a strong group-by-time interaction ($BF_{Inclusion\_Interaction} = 78.81$) between Placebo and Reconsolidation, suggesting the active treatment is more effective in the long term. In contrast, there was strong evidence that Reconsolidation-lateBAT reported more spider fear over one year compared to Reconsolidation ($BF_{10} = 777.24$), whereas Placebo and Reconsolidation-lateBAT did not seem to differ over one year overall ($BF_{10} = 0.82$).

Next, we zoomed in on whether changes in self-reported spider fear followed the behavioural changes (Hypothesis 3). As predicted, no group differences in the SPQ were found two days after treatment, before any of the groups had re-encountered spiders in the form of BATs *as indicated by an ANOVA* ($BF_{Null} = 3.44$, $BF_{Null\_Priors1} = 2.71$, $BF_{Null\_Priors2} = 9.07$)*, an ANCOVA with HBAT steps at baseline as covariate (*$BF_{Null} = 3.94$*), and pre-registered t-tests (Hypothesis 3, Prediction 1:* $BF_{Null\_Student\ t\text{-}test\_ReconsolidationVsPlacebo} = 2.51$, $BF_{Null\_Mann\text{-}Whitney\_ReconsolidationVsPlacebo} = 3.03$*, with the pre-registered default Cauchy prior distribution set to 1; Hypothesis 4, Manipulation Check 2:* $BF_{Null\_Student\ t\text{-}test\_ReconsolidationVsReconsolidation\text{-}lateBAT} = 4.39$, $BF_{Null\_Mann\text{-}Whitney\_ReconsolidationVsReconsolidation\text{-}lateBAT} = 4.12$*,with the pre-registered default Cauchy prior distribution set to 1).* Despite a strong

decrease in self-reported spider fear following the spider re-encounter two days after treatment, we did not find the predicted larger decrease in SPQ scores in the Reconsolidation compared to the Placebo group from 2 days after treatment until the 3-month follow-up ($BF_{Null} = 3.29$, $BF_{Null\_Priors1} = 2.51$, $BF_{Null\_Priors2} = 6.03$) as both groups initially showed a strong decrease in self-reported spider fear in this time period. *Thus, we also did not find the predicted group differences in self-reported spider fear between the Placebo and Reconsolidation group in the first four weeks after post-test (Hypothesis 4, Manipulation Check 1:* $BF_{Null} = 1.69$, $BF_{Null\_Priors1} = 1.68$, $BF_{Null\_Priors2} = 2.74$*).* As such, long-term benefits of Reconsolidation compared to Placebo seem to emerge later than predicted.

With regards to hypothesis 4, a RM-ANOVA suggested a group-by-time interaction between the Reconsolidation and the Reconsolidation-lateBAT group from 2 days to 4 weeks after treatment before the Reconsolidation-lateBAT group re-encountered spiders ($BF_{Inclusion\_Interaction} = 4.50$, $BF_{Inclusion\_Interaction\_CompleteCases} = 6.80$, $BF_{Inclusion\_Interaction\_Priors1} = 4.77$, $BF_{Inclusion\_Interaction\_Priors2} = 2.45$) suggesting that re-encountering spiders after treatment is indeed necessary to trigger a decrease in self-reported spider fear (Hypothesis 4).

Lastly, a pre-registered one-tailed t-test comparing SPQ scores between the two reconsolidation groups two months after their respective spider re-encounters (i.e., eight weeks after treatment for the Reconsolidation group and three months after treatment for the Reconsolidation-late BAT group) yielded anecdotal evidence for more spider fear in the Reconsolidation-lateBAT group ($BF_{10} = 1.88$, $BF_{10prior0.5} = 2.09$, $BF_{10prior1.5} = 1.21$, $BF_{10Mann\text{-}Whitney} = 1.39$). Against our predictions, the Reconsolidation-lateBAT group also reported higher SPQ scores compared to Placebo in the two months after the delayed spider re-encounter, i.e., four to twelve weeks post-treatment ($BF_{Inclusion\_Interaction} = 3.71$, $BF_{Inclusion\_Interaction\_Priors1} = 3.82$, $BF_{Inclusion\_Interaction\_Priors2} = 1.65$). Together with the previously discussed global changes in self-reported fear over the course of one year, these findings

suggest that changes in self-reported spider fear *cannot* be triggered independent of the time that has passed between the treatment and the spider re-encounters (disconfirming hypothesis 5).

Despite SPQ group means of 13.37 in the Reconsolidation group compared to 17.05 in the Placebo group at the 1-year follow-up, the benefits of the Reconsolidation group compared to the Placebo group when including the 1-year follow-up as the only outcome variable were statistically negligible ($BF_{Inclusion\_Main} = 1.13$, $BF_{Inclusion\_Main\_Priors1} = 1.22$, but $BF_{Null\_Priors2} = 1.30$; Hypothesis 6, Prediction 1), possibly due to power concerns by including one data point only. Nevertheless, the data remained most likely under the model including a main effect of group when adding baseline differences in HBAT as a covariate ($BF_{Inclusion\_Main} = 1.21$, $BF_{Inclusion\_HBATatBaseline} = 0.36$). There were no differences in self-reported spider fear between Placebo and Reconsolidation-lateBAT at the 1-year follow-up ($BF_{Null} = 3.05$, $BF_{Null\_Priors1} = 2.59$, $BF_{Null\_Priors2} = 5.46$).

Overall, these findings indicate that changes in self-reported spider fear follow the behavioural change (but also in the Placebo group) and are triggered by spider re-encounters, which should take place *within days* after the reconsolidation intervention.

S15: List of Bayes Factors for Ordinal Regressions and Frequentist Mixed ANOVAs

List of Bayes Factors for the Reported Bayesian Mixed ANOVAs for Main Outcome Variables Compared to the Omitted Ordinal Regressions as well as Exploratory Traditional Frequentist Mixed ANOVAs

**Independent variable: Group**	**Dependent variable**	**Time**	**Bayesian Mixed ANOVAs (JASP)**	**Bayesian Ordinal regressions (brms)**		**Frequentist Mixed ANOVAs (JASP)**
			Cauchy default priors	Cauchy default priors	Normal priors	
All groups	HBAT	Baseline until 3-month FU	$BF_{Inclusion\_Interaction}$ = 14.48	$BF_{Null, Interaction}$ = 0.45	$BF_{Null, Interaction}$ = 1.71	Time * Condition<br>Uncorrected: $p = 0.002$<br>Greenhouse-Geisser: $p = 0.005$
All groups	HBAT	Baseline until 1-year FU	$BF_{Inclusion\_Interaction}$ = 18.74	$BF_{Null, Interaction}$ = 2.12	$BF_{Null, Interaction}$ = 26.04	Time * Condition<br>Uncorrected: $p = 0.002$<br>Greenhouse-Geisser: $p = 0.007$
All groups	SPQ	Baseline until 1-year FU	$BF_{Inclusion\_Interaction}$ = 254.99	$BF_{Null, Interaction}$ = 0.02	$BF_{Null, Interaction}$ = 151.68	Time * Condition<br>Uncorrected: $p < .001$<br>Greenhouse-Geisser: $p = 0.007$
Reconsolidation, Placebo	SPQ	2 days to 3 months post-treatment	BF_{Null} = 3.29	$BF_{Null, Interaction}$ = 3.25	$BF_{Null, Interaction}$ = 826.13	Time * Condition<br>Uncorrected: $p = 0.142$<br>Greenhouse-Geisser: $p = 0.204$
Reconsolidation, Reconsolidation-lateBAT	SPQ	2 days to 4 weeks post-treatment	$BF_{Inclusion\_Interaction}$ = 4.50	$BF_{Null, Interaction}$ = 2.86	$BF_{Null, Interaction}$ = 3.77	Time*Condition<br>Uncorrected: $p = 0.002$<br>Greenhouse-Geisser: $p = 0.005$
Placebo, Reconsolidation-lateBAT	SPQ	4 weeks to 3 months post-treatment	$BF_{Inclusion\_Interaction}$ = 3.71	$BF_{Null, Interaction}$ = 2.89	$BF_{Null, Interaction}$ = 77.64	Time*Condition<br>Uncorrected: $p = 0.009$<br>Greenhouse-Geisser: $p = 0.024$

Note. All Bayesian mixed models control for the random effect of time in the null model. FU: follow-up. HBAT: Steps completed in the house spider behavioural approach task. SPQ: Spider Phobia Questionnaire. Normal priors: For these ordinal regressions we used the pre-registered normal prior with mean 0 and a standard deviation of 5 for fixed effects. For the SD of random effects, we used a half student-t prior with degrees of freedom equal to 3 and scale set to 10 (Gelman, 2006). As previously mentioned, these priors were too wide and did not provide realistic estimates of the variance in the data, but we want to provide the Bayes Factors for transparency. To adjust priors and to better compare these analyses to our JASP output, we used the default multivariate Cauchy prior distributions that we had also pre-registered for our mixed ANOVAs instead. Cauchy default priors: We used default multivariate Cauchy prior distributions with scale parameter set to 0.5 for fixed effects and scale parameter set to 1 for random effects (Rouder et al., 2012). With Cauchy default priors, ordinal regression models were still too complex and thus underpowered for our sample, and many BFs were unstable, i.e., the algorithm did not converge. For comparability, we also ran these analyses using traditional frequentist methods, and exploratorily provide p-values for the group-by-time interaction effects in these mixed models.

$BF_{Null, Interaction} < 1$ indicates evidence in favour of including an interaction effect into the model as opposed to the null model alone. $BF_{Inclusion\_Interaction} > 1$ indicates evidence for the model including an interaction effect compared to all other models (i.e., null and main), and $BF_{Null} > 1$ indicates evidence for the null model compared to all other models (see Data Analysis, S10). As such, BFs from the Bayesian ordinal regressions are not directly comparable with the BFs provided for the RM-ANOVAs as the JASP and brms output is not fully compatible.

As the table shows, the results for ordinal regressions using brms widely differ from Bayesian RM-ANOVAs (and their respective frequentist RM-ANOVAs) and tend to heavily favour the null model when using normal priors, even when the evidence for the inclusion of an interaction effect is strong in the RM-ANOVAs (JASP), suggesting that these priors were too wide and did not provide realistic estimates of the variance in the observed data. For further comparability, we also provide p-values for including group-by-time interaction effects based on traditional frequentist RM-ANOVAs. Results from these frequentist analyses are in line with the reported Bayesian RM-ANOVAs (using the pre-registered Cauchy priors), whereas they often differed from the omitted ordinal regressions, as would be expected.

To illustrate the discrepancies with an example, the Bayesian RM-ANOVA for a group-by-time interaction in self-reported spider fear from baseline until the 1-year follow-up suggests that the data are 254.99 times more likely under the model including an interaction effect than not, and the Bayesian ordinal regression using default Cauchy priors suggests that the data are (1/0.02 =) 50 times more likely under the model including an interaction effect as opposed to the null model, whereas with the pre-registered normal priors the data are suggested to be 151.68 times more likely under the null model as opposed to the model including an interaction effect. Even with the default Cauchy priors, the Bayesian ordinal regressions seem substantially underpowered, e.g., when the inclusion of an interaction term regarding stepwise house spider approach behaviour is suggested with substantial evidence in the Bayesian RM-ANOVAs ($BF_{Inclusion\_Interaction} = 18.74$), the Bayesian ordinal regressions may favour the null ($BF_{Null, Interaction} = 2.12$). The respective frequentist RM-ANOVA is in line with the Bayesian RM-ANOVA, suggesting that a group-by-time interaction effect should be included.

Other Descriptives

S16: Robustness of Changes in Self-Reported Fear Between the 1-Year and 3-Month Sample

There were no missing SPQ data at baseline, 2 days after treatment, and at the 3-month follow-up. At 1-year, 59 of the 69 participants filled in the SPQ again, but three participants from the Reconsolidation and Placebo group[2], as well as four participants from the Reconsolidation-lateBAT group did not return. The results were not fully robust between the full sample and the 1-year sub-sample between the Reconsolidation and Placebo groups regarding self-reported spider fear. Thus, results need to be interpreted with caution and we provide descriptives and additional information on participants who did not return.

Upon closer investigation, participants of the Placebo group who did not return (n = 3) had a lower baseline SPQ score than participants who did not return from the other two groups, whereas the three groups do not differ at baseline in the full sample and the 1-year sub-sample. Further, despite no baseline differences, the three Reconsolidation participants who did not return (n = 3) reported higher SPQ scores after treatment compared to the group mean, so it seems that lower-fear participants who took placebo did not return, whereas high-fear participants from the Reconsolidation group did not return. As participants were blind to their condition until after the 1-year follow-up, we do not believe that group assignment played a role in their decision to return 1-year post-treatment. Whereas there was a trend for propranolol to be superior to placebo 3 months after treatment, this difference is much more pronounced in the 1-year sample (n = 59), which means that our results need to be interpreted with caution.

[2] From the Reconsolidation group, two participants did not respond to our 1-year invitation and one participant moved away and stopped responding afterwards. From the Placebo group, one participant had to cancel their in-person 1-year follow-up, another participant was too busy to be scheduled, and a third participant moved abroad. All three stopped responding afterwards.

Spider Phobia Questionnaire (SPQ) Means (M) and Standard Deviations (SD) for Relevant Time Points

		Baseline	~2 days post-treatment	3-month follow-up	1-year follow-up
All available data points	Reconsolidation *M[SD] =*	n = 22 *23.27[2.71]*	n = 22 *20.32[5.08]*	n = 22 *15.50[6.98]*	n = 19 *13.37[6.30]*
(n = 59-69)	Placebo *M[SD] =*	*n = 25* *22.96[2.72]*	n = 25 *21.72[2.97]*	n = 25 *16.76[6.51]*	n = 22 *17.05[6.51]*
	Reconsolidation-lateBAT, *M[SD]=*	n = 22 *22.27[3.18]*	n = 22 *20.00[4.38]*	n = 22 *18.96[5.30]*	n = 18 *17.83[6.59]*
Participants who returned at 1-year	Reconsolidation *M[SD]=*	n = 19 *23.05[2.39]*	n = 19 *19.07[4.79]*	n = 19 *14.47[6.83]*	n = 19 *13.37[6.30]*
	Placebo *M[SD]=*	n = 22 *23.55[2.30]*	n = 22 *21.91[3.04]*	n = 22 *17.36[6.49]*	n = 22 *17.05[6.51]*
(n = 59)	Reconsolidation-lateBAT, *M[SD] =*	n = 18 *21.94[3.32]*	n = 18 *19.83[4.72]*	n = 18 *19.06[5.55]*	n = 18 *17.83[6.59]*
Participants who did not return at 1-year	Reconsolidation *M[SD]=*	n = 3 *24.67[4.73]*	n = 3 *24.33[6.11]*	n = 3 *22.00[4.36]*	NA
	Placebo *M[SD]=*	n = 3 *18.67[1.16]*	n = 3 *20.33[2.31]*	n = 3 *12.33[5.69]*	NA
(n = 10)	Reconsolidation-lateBAT, *M[SD]=*	n = 4 *23.75[2.22]*	n = 4 *20.75[2.63]*	n = 4 *18.50[4.66]*	NA

Discussion

S17: Power Analyses and Sample Size Justification

As specified in our pre-registration, we aimed to include at least 20 individuals per group who completed the first month of the study. We continued recruiting until this requirement was fulfilled and proceeded with already scheduled treatments, resulting in 69 included participants.

We used Bayesian statistics for inference and did not rely on a formal power analysis. As an initial guideline, we used G*Power (Faul et al., 2007) to estimate the minimum required sample size to detect a (very) large effect size ($f = 0.5$) with 95% power at an alpha level of 0.05, for which we would have needed a total sample of 33, i.e., 11 per group. Although this effect size was a conservative estimate compared to Soeter and Kindt's (2015) reported effect sizes, we knew that, realistically, our effects might be substantially smaller because effect sizes as large as in Soeter and Kindt's (2015) study are uncommon and effects regarding comparisons with our additional experimental group (Reconsolidation-lateBAT) were difficult to estimate. Thus, we had initially planned to use Bayesian updating with optional stopping (Rouder, 2014) until one hypothesis is favoured convincingly over another after the first 33 participants had been recruited, but as this was not practically feasible with our double-blind and longitudinal procedure, we decided against it. Hence, we opted to include the largest sample size (N = 60) that we considered feasible, knowing that we could detect significantly smaller effects than Soeter and Kindt (2015) reported.

References

Bürkner, P. C. (2017). brms: An R package for Bayesian multilevel models using Stan. *Journal of Statistical Software, 80*. https://doi.org/10.18637/jss.v080.i01

Buuren, S. van, & Groothuis-Oudshoorn, K. (2011). mice: Multivariate imputation by chained equations in R. *Journal of Statistical Software, 45*(3). https://doi.org/10.18637/jss.v045.i03

Chen, L., Valois, R. F., Toma-Drane, M., & Drane, J. W. (2005). Multiple imputation for missing ordinal data. *Journal of Modern Applied Statistical Methods, 4*(1). https://doi.org/10.22237/jmasm/1114907160

Donneau, A. F., Mauer, M., Molenberghs, G., & Albert, A. (2015). A simulation study comparing multiple imputation methods for incomplete longitudinal ordinal data. *Communications in Statistics: Simulation and Computation, 44*(5). https://doi.org/10.1080/03610918.2013.818690

Faul, F., Erdfelder, E., Lang, A. G., & Buchner, A. (2007). G*Power 3: A flexible statistical power analysis program for the social, behavioral, and biomedical sciences. *Behavior Research Methods, 39*(2). https://doi.org/10.3758/BF03193146

Gelman, A. (2006). Prior distribution for variance parameters in hierarchical models. *Bayesian Analysis, 1*(3).

Gronau, Q. F., Singmann, H., & Wagenmakers, E. J. (2020). Bridgesampling: An R package for estimating normalizing constants. *Journal of Statistical Software, 92.*

JASP Team. (2020). JASP [Computer software].

Peters, J., Visser, R. M., & Kindt, M. (2022). More than just fear: Development and psychometric evaluation of the Spider Distress Scale to assess spider fear and spider-related disgust. *Journal of Anxiety Disorders, 90*. https://doi.org/10.1016/j.janxdis.2022.102602

Rouder, J. N. (2014). Optional stopping: No problem for Bayesians. *Psychonomic Bulletin & Review, 21*(2), 301–308. https://doi.org/10.3758/s13423-014-0595-4

Rouder, J. N., Morey, R. D., Speckman, P. L., & Province, J. M. (2012). Default Bayes factors for ANOVA designs. *Journal of Mathematical Psychology, 56*(5). https://doi.org/10.1016/j.jmp.2012.08.001

Rouder, J. N., Speckman, P. L., Sun, D., Morey, R. D., & Iverson, G. (2009). Bayesian t tests for accepting and rejecting the null hypothesis. *Psychonomic Bulletin and Review, 16*(2). https://doi.org/10.3758/PBR.16.2.225

Soeter, M., & Kindt, M. (2015). An abrupt transformation of phobic behavior after a post-retrieval amnesic agent. *Biological Psychiatry, 78*(12). https://doi.org/10.1016/j.biopsych.2015.04.006

van Doorn, J., Ly, A., Marsman, M., & Wagenmakers, E. J. (2020). Bayesian rank-based hypothesis testing for the rank sum test, the signed rank test, and Spearman's ρ. *Journal of Applied Statistics, 47*(16). https://doi.org/10.1080/02664763.2019.1709053
